# Spatio-Temporal Tracking and Phylodynamics of an Urban Dengue 3 Outbreak in São Paulo, Brazil

**DOI:** 10.1371/journal.pntd.0000448

**Published:** 2009-05-26

**Authors:** Adriano Mondini, Roberta Vieira de Moraes Bronzoni, Silvia Helena Pereira Nunes, Francisco Chiaravalloti Neto, Eduardo Massad, Wladimir J. Alonso, Eduardo S. M. Lázzaro, Amena Alcântara Ferraz, Paolo Marinho de Andrade Zanotto, Maurício Lacerda Nogueira

**Affiliations:** 1 Faculdade de Medicina de São José do Rio Preto, São José do Rio Preto, Brazil; 2 Superintendência de Controle de Endemias, São José do Rio Preto, Brazil; 3 LIM 01-HCFMUSP, Faculdade de Medicina da Universidade de São Paulo, São Paulo, Brazil; 4 Forgarty International Center, National Institutes of Health, Bethesda, Maryland, United States of America; 5 Secretaria Municipal de Saúde e Higiene de São José do Rio Preto, São José do Rio Preto, Brazil; 6 Laboratório de Evolução Molecular e Bioinformática (LEMB), Departamento de Microbiologia, Instituto de Ciências Biomédicas. Universidade de São Paulo, São Paulo, Brazil; Duke University-National University of Singapore, Singapore

## Abstract

The dengue virus has a single-stranded positive-sense RNA genome of ∼10.700 nucleotides with a single open reading frame that encodes three structural (C, prM, and E) and seven nonstructural (NS1, NS2A, NS2B, NS3, NS4A, NS4B, and NS5) proteins. It possesses four antigenically distinct serotypes (DENV 1–4). Many phylogenetic studies address particularities of the different serotypes using convenience samples that are not conducive to a spatio-temporal analysis in a single urban setting. We describe the pattern of spread of distinct lineages of DENV-3 circulating in São José do Rio Preto, Brazil, during 2006. Blood samples from patients presenting dengue-like symptoms were collected for DENV testing. We performed M-N-PCR using primers based on NS5 for virus detection and identification. The fragments were purified from PCR mixtures and sequenced. The positive dengue cases were geo-coded. To type the sequenced samples, 52 reference sequences were aligned. The dataset generated was used for iterative phylogenetic reconstruction with the maximum likelihood criterion. The best demographic model, the rate of growth, rate of evolutionary change, and Time to Most Recent Common Ancestor (TMRCA) were estimated. The basic reproductive rate during the epidemics was estimated. We obtained sequences from 82 patients among 174 blood samples. We were able to geo-code 46 sequences. The alignment generated a 399-nucleotide-long dataset with 134 taxa. The phylogenetic analysis indicated that all samples were of DENV-3 and related to strains circulating on the isle of Martinique in 2000–2001. Sixty DENV-3 from São José do Rio Preto formed a monophyletic group (lineage 1), closely related to the remaining 22 isolates (lineage 2). We assumed that these lineages appeared before 2006 in different occasions. By transforming the inferred exponential growth rates into the basic reproductive rate, we obtained values for lineage 1 of R_0_ = 1.53 and values for lineage 2 of R_0_ = 1.13. Under the exponential model, TMRCA of lineage 1 dated 1 year and lineage 2 dated 3.4 years before the last sampling. The possibility of inferring the spatio-temporal dynamics from genetic data has been generally little explored, and it may shed light on DENV circulation. The use of both geographic and temporally structured phylogenetic data provided a detailed view on the spread of at least two dengue viral strains in a populated urban area.

## Author Summary

Most of the molecular phylogeny studies of dengue fever, an important public health problem, use convenience samples for their analysis, and they do not evaluate the spatial and temporal features involved in the spread of the different serotypes (and genotypes) circulating in urban settings during an outbreak. Our study describes the patterns of spread of different lineages of dengue 3 virus circulating in a medium-sized city from Brazil, and we also analyzed the dynamics and microevolution of the disease during the 2006 outbreak. We used both geographic and temporally structured phylogenetic data, which provided a relatively detailed view on the spread of at least two dengue viral lineages circulating in an urban area. The pattern of dengue virus circulation might be similar to many other settings all over the world, and the information provided by our study can help a better understanding of dengue outbreaks, providing important information for public-health systems. We could identify at least two lineages, which were introduced in different occasions. They circulated and spread at different rates within the city, and this differential spread and the role of socioeconomic features in this phenomenon are discussed.

## Introduction

The genus *Flavivirus* includes 53 arthropod borne viruses that can cause severe encephalitis, hemorrhagic fever and febrile illness in humans [1]. Dengue viruses (DENV), Saint Louis Encephalitis virus (SLEV), and Yellow Fever virus (YFV) belong to this genus and are important public health issues in most tropical and subtropical countries [2]. Dengue is the most common arboviral infection all over the world [3]. Like other flaviviruses, dengue virus has a single-stranded positive-sense RNA genome of ∼10,700 nucleotides that is surrounded by a nucleocapsid and covered by a lipid envelope with viral glycoproteins. The RNA genome contains a single open reading frame (ORF) flanked by two untranslated regions (UTRs 3′ and 5′). The single ORF encodes a precursor polyprotein, which is co- and post-translationally cleaved into three structural (C, prM and E) and seven nonstructural (NS1, NS2A, NS2B, NS3, NS4A, NS4B, NS5) proteins [4]. The disease is caused by four antigenically distinct virus serotypes (DENV 1–4) and each serotype harbors phylogenetically defined genotypes [5] that have been experiencing massive bursts of genetic diversity as a consequence of the exponentially increasing human population during the last 200 years [5],[6],[7].

Dengue infection may be asymptomatic and lead to undifferentiated fever, dengue fever (DF) or evolve to more serious conditions (dengue hemorrhagic fever (DHF) or dengue shock syndrome (DSS)) [3],[8]. DF is an acute febrile viral disease that is characterized by headaches, biphasic fever, skin rash, retro orbital pain, leukopenia, thrombocytopenia and lymphadenopathy [3]. DHF is characterized by high fever, hemorrhagic manifestations and signs of circulatory failure. Patients presenting such symptoms may develop hypovolemic shock, leading to DSS, which can be fatal [8]. Outside Africa, the disease is transmitted mainly by the *Aedes aegypti* mosquito, which is widely distributed and established in all tropical countries and subtropical countries. Nearly three billion people are at risk of infection by DENV [9]. Brazil was responsible for approximately 94.5% of the reported dengue cases in Central and South America and 60% all over the world in 2007. Moreover, until the 39^th^ epidemiological week, which started in September 23^rd^ 2007 and finished in 29^th^ 2007, 481.316 cases of DF (out of a population of approximately 186 million people, www.ibge.gov.br/english/) were reported along with 1076 DHF manifestations [10]. At the same period, São Paulo State with 21% of the Brazilian population was responsible for 17% of the cases (82.684). The impact of the disease is very heterogeneous in the State: the city of São José do Rio Preto – included in our study - reported 12% (9.331) of the occurrences in the State having only 1% of its population [11]. Even before the 2006 outbreak, dengue was endemic in São José do Rio Preto [12]. Many molecular phylogeny studies addressed particularities of the dynamics of the different dengue serotypes [6],[7],[13],[14],[15],[16],[17],[18],[19],[20],[21],[22],[23],[24],[25]. However, there is still a need to study particular outbreaks in single urban settings at a fine-grained spatio-temporal scale. In the present work we describe the pattern of spread of distinct lineages of DENV-3 virus circulating in São José do Rio Preto, São Paulo, Brazil during the 2006 outbreak and analyze the dynamics and microevolution during the outbreak.

## Materials and Methods

### Study site

The city of São José do Rio Preto (SJRP) is on the northwestern region of São Paulo State, Brazil (20°49′11″ S e 49°22′46″ W), with a total area of 434,10 Km^2^ and an urban area of 96,81 Km^2^. The estimated population in 2007 was 424,114. SJRP has a tropical climate with a mean annual temperature of 25°C and mean rainfall of 1410 mm concentrated in the summer months. The city has development indexes comparable to those of developed countries and its economy encompasses industry, services, commerce and agro-business. The urban area of the municipality is divided in 432 census tracts. The census tracts comprise 300 homes in areas defined by the *Instituto Brasileiro de Geografia e Estatística — IBGE* (Brazilian Institute of Geography and Statistics) to optimize the collection of data sets during census. Although SJRP was infested by the *Aedes aegypti* in 1985, only imported dengue cases where reported until 1989. Human to human DENV-1 transmission was first observed in 1990. From that date, dengue cases have been reported every year, with the exception of 1992 [12] and DENV-2 and DENV-3 were introduced in 1998 and 2006.

### Geo-coding

Geo-coding of autochthonous dengue cases was done using ArcGIS 9.0 (Environmental Systems Research Institute, Inc.). The geographic position of each patient was assumed to be the latitude and longitude of their postal code (zip code) obtained from sampled patient address records provided by the municipality of São José do Rio Preto.

### Sample collection

Blood samples from patients presenting acute febrile illness, with or without hemorrhagic manifestations, infection with sudden start, nausea, vomit, diarrhea, symptoms of DF and DHF were collected for *Flavivirus* testing in the municipal health units and hospitals, upon informed consent. This study was approved by the Ethical Review Board of the Faculdade de Medicina de São José do Rio Preto and blood collection was performed upon Written Informed Consent.

### cDNA synthesis and sequencing

The blood samples were centrifuged and the viral RNA was extracted from the serum with the QIAmp viral RNA mini kit (Qiagen) according to the manufacturer's instructions. The first RT-PCR was performed using Flavivirus generic primers based on the non-structural protein 5 (NS5), which is a conserved region in dengue viruses and would detect most of the circulating dengue virus in Brazil in a single PCR reaction. In the second PCR, nested assays based on multiplex or conventional systems were used with species-specific primers for virus identification [26]. The forward FG1 (5′TCAAGGAACTCCACACATGAGATGTACT3′) and reverse FG2 (5′GTGTCCCATCCTGCTGTGTCATCAGCATACA3′) primer set anneals to the NS5 gene, producing amplicons of approximately 958 bp [27]. A specific inner primer for DENV-3 (5′TTCCTCGTCCTCAACAGCAGCTCTCGCACT3′) produced amplicons with 659 bp [26]. The fragments were purified from PCR mixtures and sequenced with the BigDye v3.1 Terminator (Applied Biosystens, Foster City, CA, USA) using the forward FG1 primer and the reverse DENV-3 primer in an ABI377 automated sequencer (Applied Biosystenss, Foster City, CA, USA). The products were aligned with Accelrys Gene 2.0 (Accelrys Software Inc. 2006).

### Phylogenetic reconstruction

In order to type the sequenced samples, 52 reference sequences including representatives of the 4 serotypes were hand-aligned in Se-Al version 2.0a11 program (data available from authors upon request). The dataset generated was used for phylogenetic reconstruction with the maximum likelihood criterion using a genetic algorithm method implemented in the program GARLI version 0.95 [28] that estimates simultaneously the best topology, branch lengths and the best values for the parameters for the General Time Reversible (GTR) model of nucleotide evolution with Gamma-distributed variable rates and invariant sites (GTR+Γ+I). One hundred independent random runs were conducted with GARLI and the tree with highest likelihood was subsequently used as input for further topological optimization with PAUP v.4.0b10 [29], since both GARLI and PAUP calculate the same likelihood score for a tree under the same model. Support for the topology was sought after 100 bootstrap replicates with GARLI.

### Phylodynamics of dengue 3

The best demographic model among: (*i*) constant population size, (*ii*) exponential population growth and, (*iii*) logistic population growth for the data, the rate of growth (*r* = *Ne.g*) (*i.e.*, the effective number of transmission events times the generation time of the pathogen), rate of evolutionary change (μ) (subs/site/year) and, the Time to Most Recent Common Ancestor (TMRCA) were estimated from the Coalescent using Bayesian inference with a Markov Chain Monte Carlo (MCMC) search method available in BEAST v1.6 [30]. Sequences were dated according to the day of sampling and the MCMC were run until the effective sampling size (ESS) for each parameter converged at values above 100. The confidence intervals for each parameter were given by the 95% highest probability density (HPD). The data was analyzed using a relaxed molecular clock (uncorrelated lognormal) under the constant, exponential and logistic demographic models. Because the priors were not necessarily the same, demographic models were compared by calculating the Log 10 of the Bayes factor using the harmonic mean without smoothing of the sum of the likelihoods for the coalescent and tree obtained during each MCMC for each model with TRACER v1.6 program [30].

### Analysis of spatio-temporal dispersion patterns

First, to examine whether the samples were overly spatially or temporally structured we generated matrices of distances between the samples and compared those matrices. The matrices of distances show how each sample is relatively close to all others, considering separately their date of collection, their place of collection and their genetics. Corrected genetic distances were obtained with PAUP using the values for the GTR+Γ+I model found for the maximum likelihood tree inferred with GARLI. Geographic distances were measured along straight lines (using geographic information system) between samples and temporal distances were the interval between sample collections. The null hypothesis of no association between the genetic and geographic and temporal distances was assessed using partial Mantel tests [31]. Statistical distributions were generated by a Monte Carlo method randomising rows (and corresponding columns) in the matrix of phylogenetic distances 1,000 times, and calculating for each one of these permutations the partial correlation coefficient between the two matrices, controlling for the third (geographic) matrix. The one-tailed probability of a type I error (*i.e.*, rejection of a true null hypothesis) was taken as the proportion of correlation coefficients sorted in ascending order that were higher than or equal to the obtained correlation coefficient. We accepted probabilities below α = 0.05 as statistically significant. Second, to obtain the putative spatio-temporal pattern of spread of dengue in SJRP we applied a simple algorithm that checks all samples (but the first) in a temporal sequence, and would attribute the linkage of ancestry to the (temporally) previous sample with higher genetic proximity (hence, let's call it “nepotistic algorithm”). No obvious important geographic barriers for the circulation of the vector or host were identified within the area studied; hence we did not add any friction/cost to the movement of viruses into the algorithm. We did apply instead a limit of 0.00739 substitutions/site/year that we considered as the genetic distance that could have been generated by a virus replicating during 100 days at a rate of 10^−4^ substitutions/site/year. Therefore, if one sample was not closer in distance than this value to any of its chronologically previous samples, it was assumed that this sample resulted from another virus introduction in the locality.

### Calculation of the Basic Reproduction Number (R_0_) for the 2006 SJRP dengue epidemics

Because each viral sequence was obtained from a distinct patient, nodes in the virus gene genealogy can be assumed as transmission events in the human population. Therefore the basic reproductive rate of a pathogen (R_0_) during an epidemics can be estimated as R_0_ = 1+*D*(ln2/*t*d) [32], where *D* is the mean time of infectiousness (*i.e.*, 7 days for virus shedding in humans) [33] and *t*d is the doubling time of the epidemics and, since the growth rate (*r*) obtained from the viral phylodynamics equals ln2/*t*d [30], we estimated R_0_ as 1+*Dr*. However, the monotonic-increasing population models (exponential en logistic) available in BEAST do not account properly for fluctuating dynamics, such as that observed during the studied outbreak, possibly affecting the growth rate estimates using these models. This was further substantiated by the fluctuating nature of the Bayesian skyline (BSL) plot for dengue that showed rapid increase at the onset of the epidemic phase, followed in time by a reduction of cases at the end of the outbreak. Therefore, we also used for comparison an alternative way of estimating R_0_, based on the growth phase of the epidemics alone, by deriving the force of infection at the increasing phase of the BSL as follows. The normalized median of the Bayesian estimates from the sequences analyzed, *y(t)*, was fitted to a continuous logistic curve according to the following model:
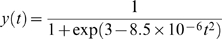
(1)


From equation (1) it is possible to estimate the force of infection for the data [34],[35]:
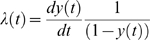
(2)where,
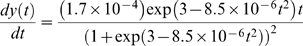
(3)


The Basic Reproduction Number, R_0_, was estimated from the average force of infection, calculated from equation (2) by its equality to the number of new cases per time unit per susceptibles, according to a previous study [33]:
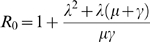
(4)where, *μ* is the mosquito mortality rate and *γ* is the recovery rate of viraemia in humans. The mosquitoes mortality rate is the rate by which moquitoes die, on average, in each unit of time and is the inverse of the average life expectancy of each specific mosquito population. It varies from place to place and in the same place it also varies with environmental conditions, like temperature, rain pattern and othe climatic variables. We used the mosquito mortality rate of to 2.23×10-2/day previously estimated for SJRP [33].

## Results

### Dengue characterization and sequencing

We obtained 399 bp-long sequences of a portion of the NS5 gene from viral genomic RNA amplified directly from the blood of 82 patients from 198 samples collected in the city of São José do Rio Preto for a period of 174 days, from January 12 to June 5^th^ of 2006, which covered the zenith of the outbreak (*i.e.*, above 1000 cases per 100,000 inhabitants) in April 2006 (GenBank accession numbers from EU715692 to EU715773). We were able to geo-locate 46 patients based on the addresses of their residences, from the cohort of 82 patients. The reason for not geo-coding the other 36 patients was the lack of their complete home address.

### Dengue typing and origin

**Figure 1:**
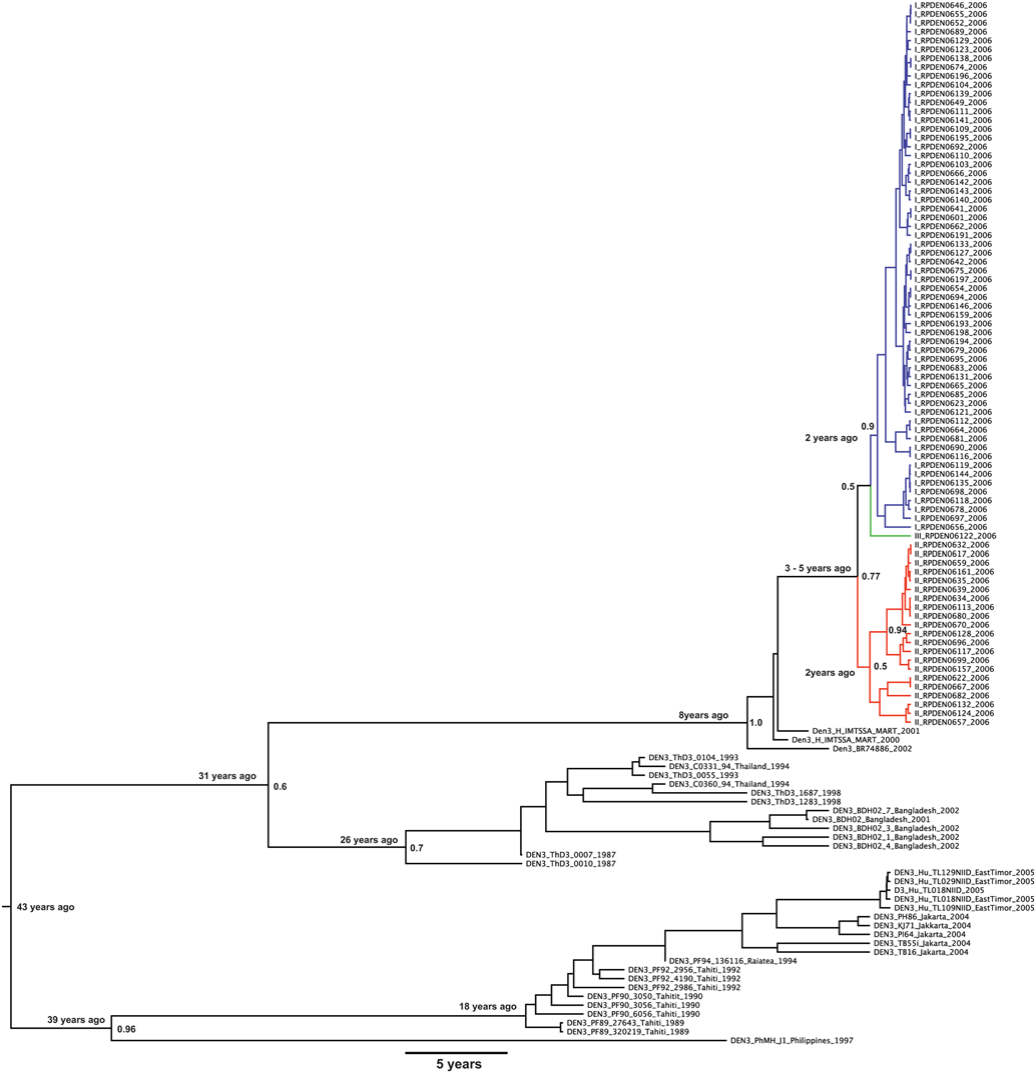
Maximum clade credibility (MCC) tree for 131 Dengue 3 isolates. MCC tree for 131 Dengue 3 isolates from several places in World, indicating that the origin of the 60 lineage 1 samples (with 90% posterior probability support) from viruses from the lineage 2 (with 77% posterior probability support). The close-related BR47886 sampled in 2002 in Brazil and the H IMTSSA sampled in 2000 in Martinique had a basal position in relation to the SJRP lineages (with 100% posterior probability support).

By aligning the 82 sequences with the 52 references we generated a 399-nucleotide-long dataset (without gaps) with 134 taxa. Preliminary phylogenetic analyzes, including the 4 serotypes indicated that all samples were of serotype 3 (data not shown). Furthermore, all of our samples nested within DENV-3 and were closely related to strains circulating in the isle of Martinique in the Caribbean in 2000 and 2001 and, to the DENV-3 strain Den3_BR74886 circulating in Brazil in 2002 (Figure 1). Moreover, Figure 1 indicated that, given the reference samples included in our analyses, the South American lineages were more related to lineages circulating in South East Asia. Another important finding was that 60 DENV-3 from SJRP formed a monophyletic group (lineage 1, shown in blue) with 90% posterior probability, which were closely related to the remaining 22 isolates that did not form a clear monophyletic cluster (lineage 2 and 3, shown in orange) that had a more basal position in the tree and that intermingled with the other South American references available from Martinique and Brazil.

### DENV-3 dynamics in SJRP

**Figure 2:**
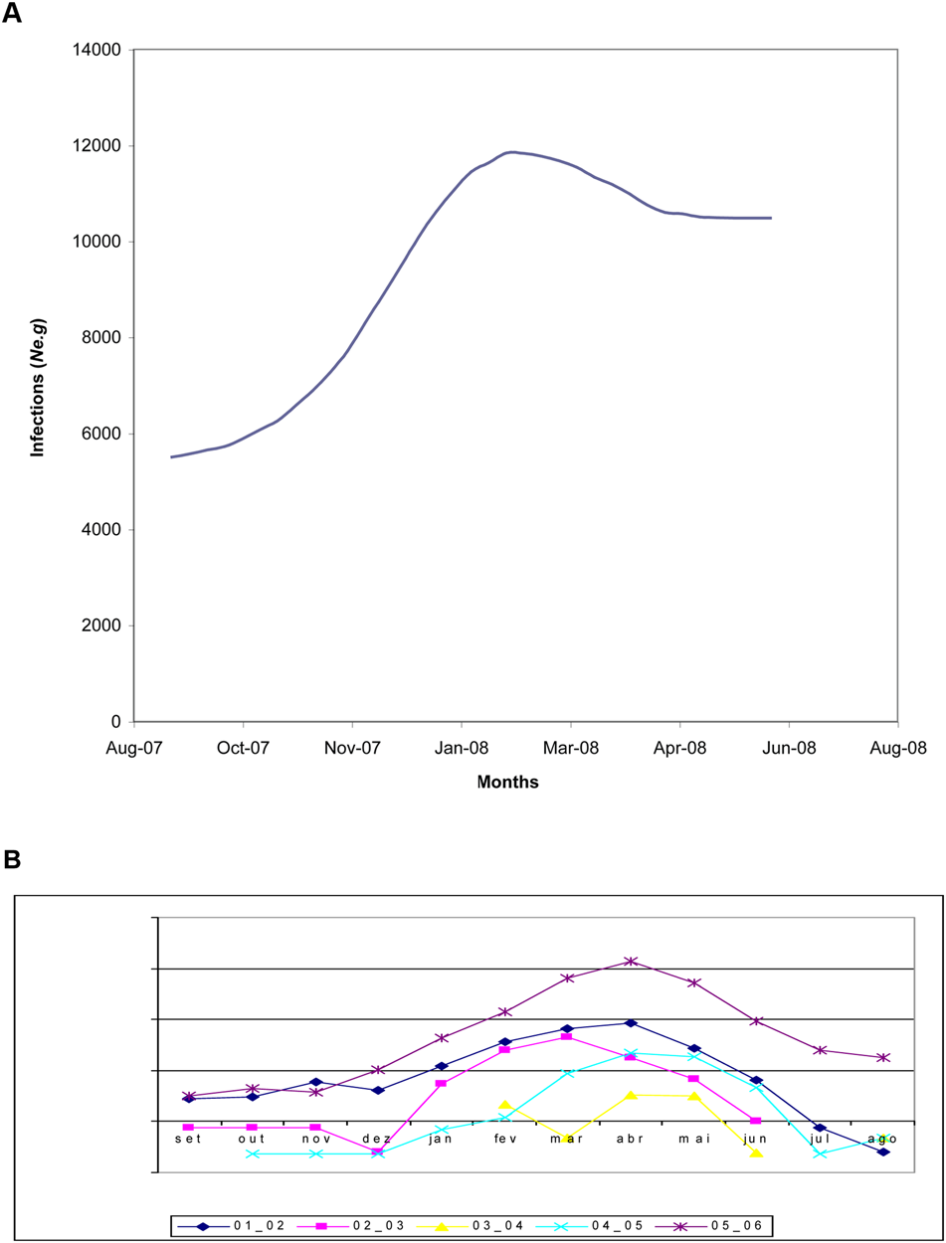
Bayesian skyline (BSL) plot and number of dengue reported cases. A) Bayesian skyline (BSL) plot of the virus genealogy-based estimate of the number of new infections (presented as *Ne.g*) indicated as the median for 82 DENV-3 isolates showing the increase from 180 to 120 days before the last sampling (from present day 0 or day of the last sample taken to the past), which matches with uncanny precision the rise in number of reported cases per 100,000 inhabitants from December of 2005. Apparent differences in overall population sizes are due to both the fact that the BSL shows accumulated number of new infections and to scaling problems or misreport. B) Number of Dengue reported cases in SJRP during the seasons of 2001–2002 (01_02), 2003 (02_03), 2004 (03_04), 2005 (04_05) and 2006 (05_06). It is noticeable that the maximum number of reported cases in 2006 happened in April, when the zenith of the epidemics, determined by the Bayesian skyline plot, was around February.

**Figure 3:**
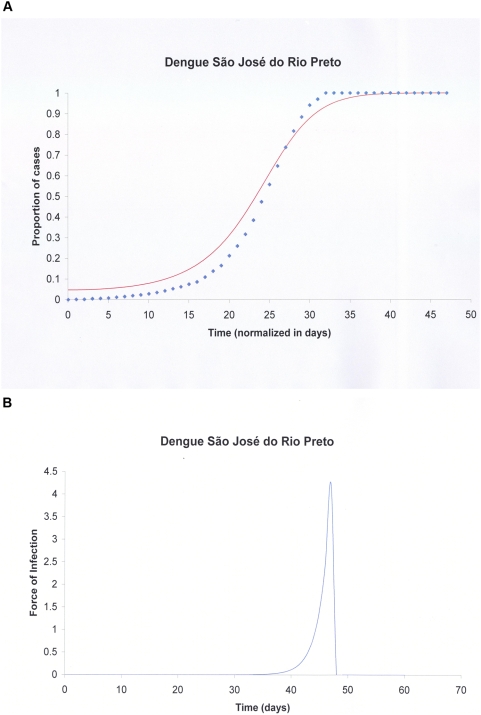
Logistic fitting of the Bayesian skyline plot and force of infection. A) the normalized median of the Bayesian estimates from the sequences analyzed, *y(t)*, fitted to a continuous logistic curve according to equation (1). Diamonds represent the data and the continuous line the fitted function. The period of time entails the epidemics during the fat growing phase during January and February of 2006. B) Force of infection calculated from the fitted model in A) using equation (2).

Our demographic analysis using a Bayesian skyline prior with BEAST generated a maximum clade credibility (MCC) tree with dated tips and internal nodes that indicated that both lineages split 1 to 3 years before the last sample was collected in SJRP (Figure 1). Moreover, since there was no sustained DENV- 3 epidemic transmission during this entire period in SJRP, we assumed that these lineages appeared before the 2006 season and that distinct lineages were possibly introduced into the city on different occasions. As a consequence, the major lineages were treated as distinct viral populations during subsequent demographic analyses. Since, most lineages shown in orange in Figure 1 had a common ancestor with 77% posterior probability , they were grouped into a single group (lineage 2) for the sake of demographic analyses. For both groups of samples there was significant population growth initiating 6 months before the last sampling, which matches quite well with reports increasing above 10 cases per 100,000 in December 2005 (Figure 2). Moreover, for both DENV-3 lineages, the constant population size model was rejected (for lineage 1, Log 10 Bayes factor>250; lineage 2, Log 10 Bayes factor>146). Although the signature of BSL in Figure 2 is clearly logistic until around the zenith of the outbreak, the logistic model did not out-perform the exponential growth model for both lineage 1 (Log 10 Bayes factor = 23.9) and lineage 2 (Log 10 Bayes factor = −4.436), in the latter case even if the logistic model had a higher Bayes factor (146.251) when compared to the exponential (150.686), there was no significant improvement by including additional logistic parameters to describe the data. Critically, as indicated by the HPD, the growth rate (*r*) for both lineages were significantly above zero for both, lineage 1, *r* = 0.0752 (with 95% HPD from 6.96E-5 to 0.258) with an ESS of 107.75 for 150,400,000 states, and lineage 2, *r* = 0.0182 (with 95% HPD from 1.627E-4 to 0.0391) with an ESS of 1239.444 for 325,600,000 states. By transforming the inferred exponential growth rates into the basic reproductive rate, we obtained values for lineage 1 of R_0_ = 1.53 (with 95% HPD ranging from above 1 to 2.8) and values for lineage 2 of R_0_ = 1.13 (with 95% HPD ranging from above 1 to 1.3). Although we rejected the logistic model, the basic reproductive rate obtained from the logistic growth rate values (data not shown) for lineage 1 was R_0_ = 3.765 (with 95% HPD ranging from above 1 to 9.554) and for lineage 2 was R_0_ = 3.093 (with 95% HPD ranging from above 1 to 8.896). These data meant that the rate of growth was almost 50% higher for lineage 1 at exponential growth but only 17% higher under the logistic model. Under the exponential model the most recent common ancestor MRCA of lineage 1 dated 2 years before the last sampling (with 95% HPD ranging from 6 months to 3 years) and lineage 2 also dated 2 years before the last sampling (with 95% HPD ranging from 6 months to 5 years). In sum both lineages appear to have similar growth patterns with a trend of increased rate of growth (and higher R_0_) for lineage 1 strains. One serious limitation of the former approach was that the monotonic-increasing models used in BEAST (logistic and exponential) may have not captured the true fluctuating dynamics of the epidemics, since both fail to detect the decrease in numbers of new infections after the Zenith of the outbreak. Therefore we also used other methods [33],[34],[35],[36]. The logistic fitting of the Bayesian skyline plot inferred by MCMC from viral genealogies for *y(t)* is shown in Figure 3A. The Basic Reproduction Number, R_0_, was estimated from the average force of infection (Figure 3B), calculated from equation (2) and equal to 0.17 new cases per time unit per susceptibles, according to the method previously proposed in a study [33]. From equation (4) the basic reproduction number (R_0_) obtained was 2.45. The data suggested that there was a good match among values obtained directly from the growth rate estimated with BEAST, the one found using the force of infection and, the epidemiological estimates of 3.36 previously estimated [37] from the cases' doubling time.

### Spatio-temporal distribution of DENV-3 lineages in SJRP

**Figure 4:**
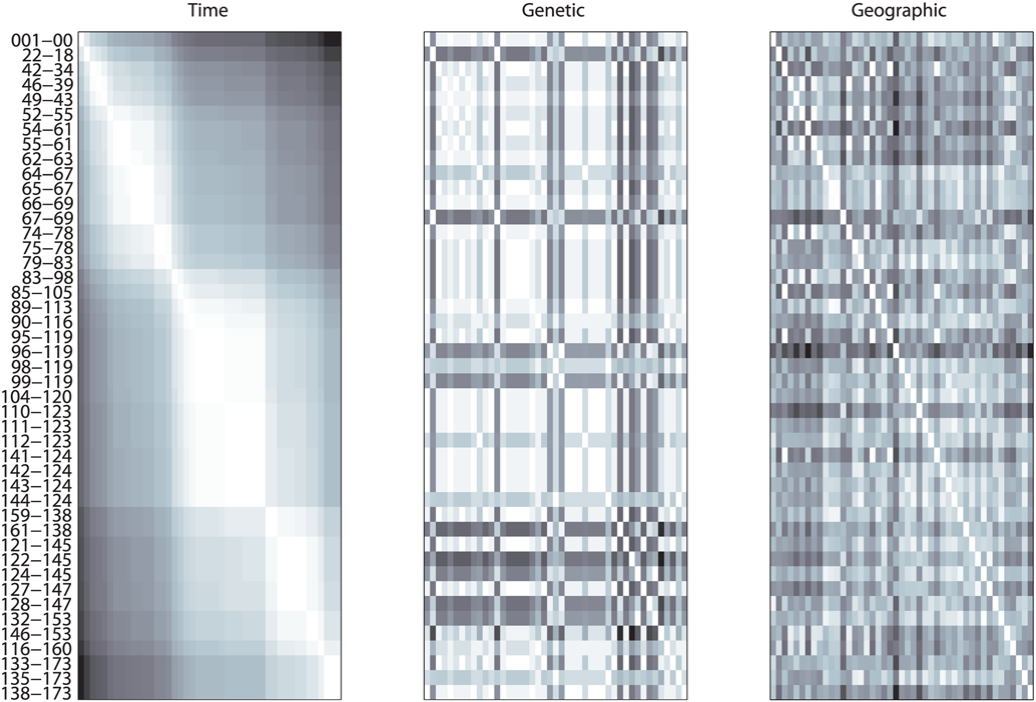
Symmetrical matrices. Symmetrical matrices representing: A) temporal, B) genetic and C) spatial distances among the 46 samples. For easy visual comparison, numbers were substituted by a scale of colours that ranges from white (lower value) to black (higher value). The samples were organized temporally as indicated by the gradual pattern observed in matrix A), and the colours of the names of the samples are meant to facilitate the comparison with the results.

By visually inspecting the temporally sorted distance matrices shown in Figure 4, we noticed some genetically similar ‘blocks’ (bright patches in the second matrix) following the temporal gradient (first matrix), but intercalated with “dark” lines representing lower genetic proximity. This intercalation of genetically distant samples seems to be responsible for preventing an overall statistical association and was due to the distinct lineages co-circulating in SJRP during the outbreak. There was also no spatial association between samples (third matrix) in any noticeable way when compared with the other matrices. These visual observations were confirmed by the statistical analyses, Spearman correlation between the other ones was very low (r = 0.06 with geographic, and r = 0.01 with temporal) and non-significant (<0.05 as obtained by Mantel method with 1,000 interactions, [31]).

**Figure 5:**
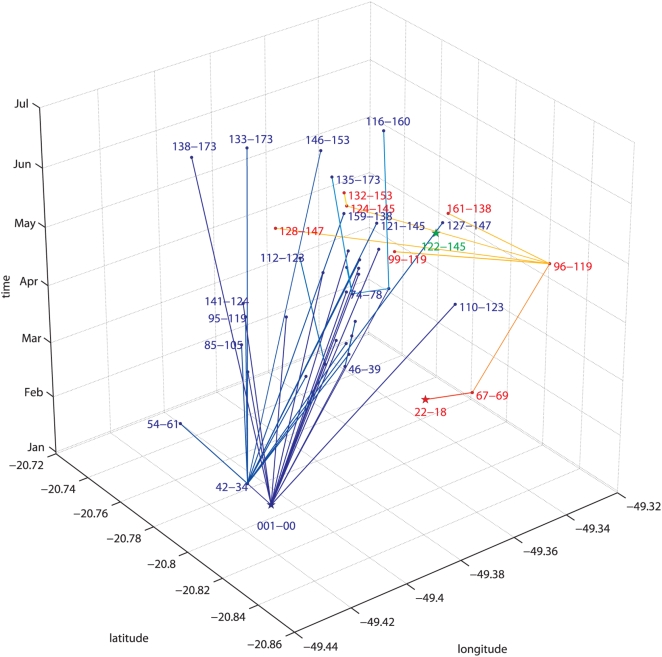
Estimated routes of dispersion of dengue as obtained by the “Nepotistic algorithm”. The basis of the three dimensional graphic represents the geographic coordinates of the positions where the sample were collected, while the vertical axis represents the months of the sampling. Each independent lineage starts with a “star” (oldest sample of the lineage) and the connections to the other samples (names are displayed as close to point as possible) are done with lines, whose width is proportional to the genetic relatedness between the samples. The three lineages represented are: (*i*) green the more recent, with only one sample, the 122-145, (*ii*) red or “South-eastern” lineage (starting with 22-18, that turns to yellow with more recent “ancestry” samples) and (*iii*) blue, or North-western” lineage 1 (starting at 01-00, and that gets lighter as for the previous one).

The analyses with the “nepotistic algorithm” suggested the existence of at least three possibly independent introductions of strains from lineages 1 and 2 into SJRP. The three lineages correspond well to the tree shown in Figure 1. The proposed spatial-temporal associations were represented in three dimensions in Figure 5, which explained the lack of overall statistical association between the correlation matrices. The three main virus introductions appeared as genetically-similar blocks intercalated with more distant rows in the genetic distance matrix of Figure 4. In fact, we can see that the “predominantly darker lines” coincide with the lineages names shown in red in Figures 4 and 5. A closer inspection also revealed that, similarly to what was observed for to the “blue” samples of lineage 1, the genetic distances between the “red samples” were low among themselves. The branching pattern of the spatio-temporal tree (Figure 5) generated with the “nepotistic algorithm” also explained why there was no correlation between geographic distance matrix and the other distances. This was because the spatial dispersion of the virus starts in three different points and does not appear to follow a single centrifugal pattern.

**Figure 6:**
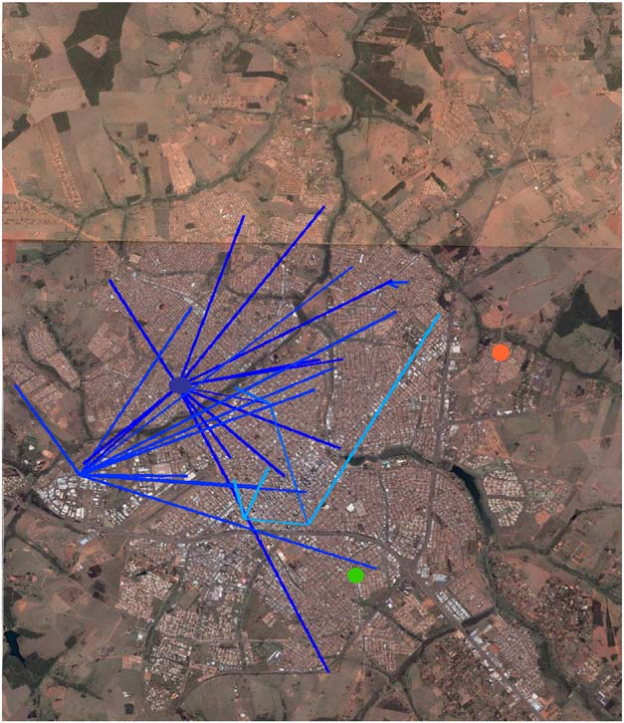
Route of viral dispersion - I. Estimated route of dispersion of the “North-western”, departing from the putative ancestor (blue dot and dispersing to other places where dengue cases were recorded - lines with more recent nodes tends to the lighter tones) projected on the aerial image of the city of São José do Rio Preto (SJRP). The sample of the third viral introduction is also shown in a green dot. (Background image obtained from Google Earth 4.2.0181.2634 – download date: September 16th 2007).

**Figure 7:**
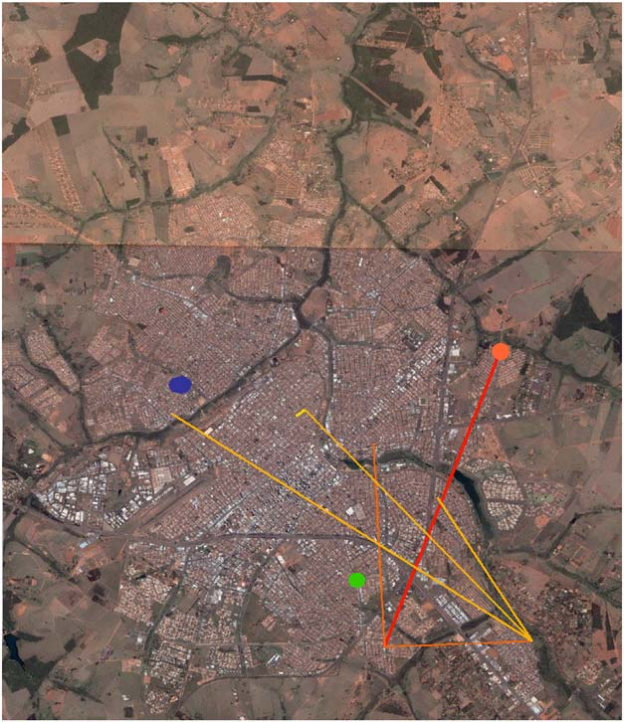
Route of viral dispersion - II. Estimated route of dispersion of the “South-eastern”, departing from the putative ancestor (orange dot) and dispersing to other places where dengue cases were recorded - lines with more recent nodes tends to the yellow tones) projected on the aerial image of the city of São José do Rio Preto. The sample of the third viral introduction is also shown in a green dot. (Background image obtained from Google Earth 4.2.0181.2634 – download date: September 16th 2007).

The “North-western” component (lineage 1) shown in blue in the spatio-temporal trees in Figures 5 and 6, included the earliest samples from January 12^th^. It was also the most prevalent (36 samples), and was the more long-lasting, encompassing also the 5 more recent samples: *‘138-173’*, *146-153’*, *‘116-160*’, *‘133-173’*, *‘135-173’*, and *‘138-173’*, collected between June 14^th^ and July 4^th^. The connections between lineage 1 samples averaged 3.25 kilometres (ranging from 15 meters to 7.23 kilometres). The average of speed of the propagation was around 67.3 meters per day, ranging from 18 centimeters/day (since two samples (*‘95-119’* and *‘42-34’*) were collected only 15 meters apart) to a maximum speed of 428.8 meters/day. Moreover, some lineage 1 samples seem to be the possible source nodes of many other samples (the names of these samples and the number of generated links are: ‘*01-00*’: 16; ‘*42-34*’: 12; *‘64-67’*: 2; *‘90-116’*:2; *‘98-119’*:2). The “South-eastern” samples bundled into at least lineage 2 shown in red in the spatio-temporal tree (Figures 5 and 7), had lesser components (8 samples). Its recorded activity ranged from January 30^th^ (*‘22-18’*) to June 14^th^ (*‘132-153’*). The average length of its connections was 4.7 kilometres (min: 0.2, max: 8.7 kilometres), at an average speed of 152 meters per day (min: 28 meters/day; max: 311 meters/day). The ‘122-145’ (Figures 5, 6, and 7) would constitute another entry of dengue into SJRP.

### Socioeconomic features

**Figure 8:**
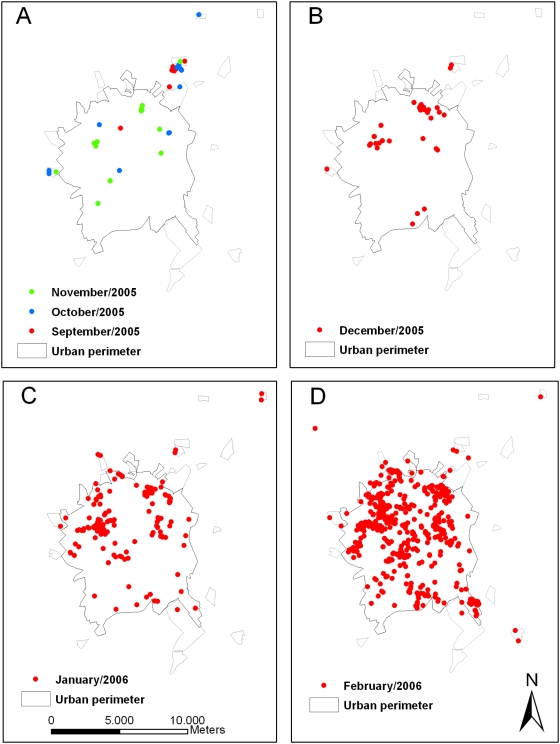
São José do Rio Preto map. São José do Rio Preto map with urban census tracts, irregular development areas and autochthonous dengue cases reported and confirmed by the Surveillance System from September to November (A), December (B), January (C) and February (D). The areas outside the urban perimeter are irregular development areas with urban characteristics, but with inadequate sanitation infrastructure and lowest socioeconomic conditions in comparison to urban census tracts. There is a cluster of cases in one of these irregular areas in the North Zone of the city (Santa Clara) in September 2005 and a spread of the transmission to the rest of the urban perimeter (A).

**Figure 9:**
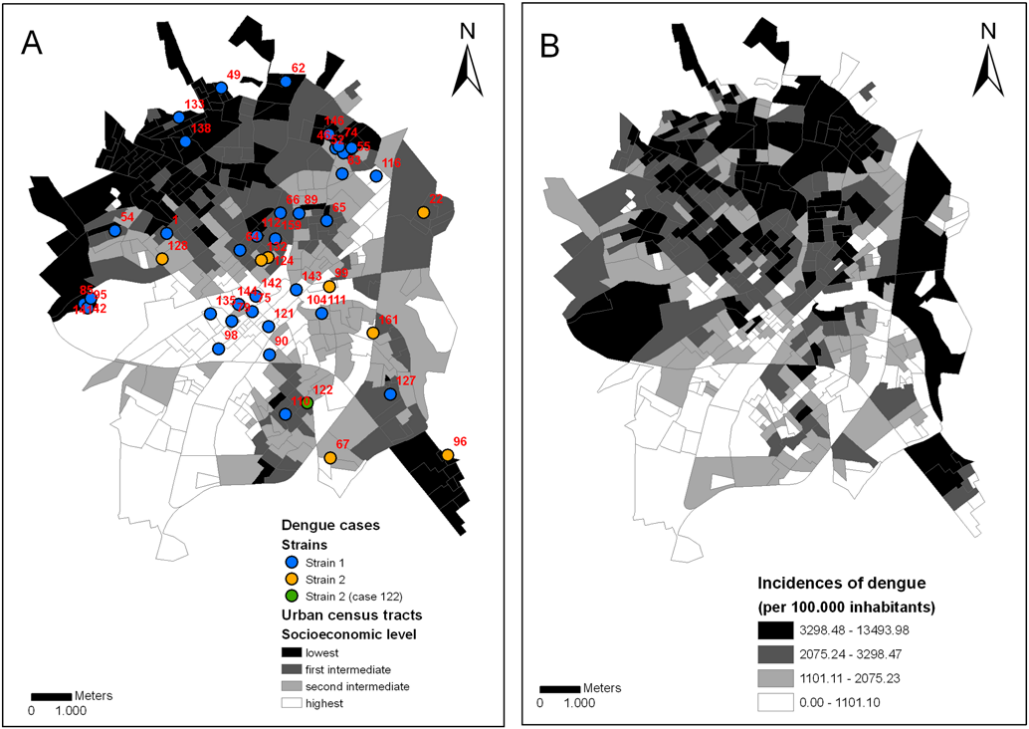
Urban census tracts. Urban census tracts (*i.e.*, continuous and homogeneous areas comprising 300 buildings on average, IBGE 2002) according to socioeconomic levels (quartile) and dengue cases with molecular analysis according to strains from January 2006 to June 2006 (A); urban census tracts according to incidence coefficients of dengue cases (quartile) reported to the Surveillance System from September 2005 to August 2006 (B).

Figure 8 represents the autochthonous dengue cases confirmed by the Surveillance Service from September 2005 to February 2006. In September 2005, there were dengue cases within an irregular residential area (Santa Clara) in the northern area and outside the urban perimeter, which lacks proper basic sanitation. During the following months, there were several additional cases in the neighborhood and the disease spread into other areas of the urban perimeter. The molecular characterization of the circulating strains identified at least three different viral introductions 01-00, 22-18 and122-145. The first event was at Eldorado, a neighborhood with low socioeconomic levels. Other cases occurred in areas with different socioeconomic levels (Figures 5, 6, and 9). There was a cluster at Gonzaga de Campos (cases 42, 85, 95 and 141), a working-class neighborhood with low socioeconomic levels, flanking the main industrial zone of the municipality. A series of cases linked to case 42 spread among areas with different socioeconomic levels (Figures 5, 6, and 9). Case 22-18, which led to other cases in lineage 2, with the exception of case 122-145, that also occurred in São Deocleciano, an area with low socioeconomic level. There was a relation among the socioeconomic level of the census tracts and the incidence coefficients based on the cases reported by the Surveillance System (Table 1), despite an even distribution of the RT-PCR positive cases among different census tracts (Table 1 and Figure 9). Approximately 44% of the census tracts of the lowest socioeconomic level were in the highest quartile of incidences, but only 5.5% of the census tracts of the highest socioeconomic level were in the same situation.

**Table 1:** Relation among the socioeconomic level of the census tracts and the incidence coefficients.

Socioeconomic levels of census tracts (quartile)		Low	Inferior Intermediate	Superior Intermediate	High	Total
Molecular analysis (%)*		13 (28.9)	12 (26.7)	9 (20.0)	11 (24.4)	45 (100.0)
Census tracts according quartile of IC (%)**	1°	10 (9.3)	15 (13.9)	29 (26.9)	55 (50.5)	109
	1°–2°	14 (13.0)	27 (25.0)	35 (32.4)	32 (29.4)	108
	2°–3°	3734.3)	28 (25.9)	27 (25.0)	16 (14.7)	108
	3°–4°	47 (43.5)	38 (35.2)	17 (15.7)	6 (5.5)	108
	Total	108 (100.0)	108 (100.0)	108 (100.0)	109 (100.0)	433

*: Qui-squared test for non-significant adherence: χ^2^ = 0.778; p = 0,8548.

**: Qui-squared test for significant independence: χ^2^ = 101.679; p<0.0001.

## Discussion

Inferences based on sequence data of spatio-temporal dynamics, including the speed and direction of virus propagation, have been little explored until recently [38]. Nevertheless, they can help to better understand dengue outbreaks, providing useful information for public-health systems. In this study we showcased the use of both geographic and temporally structured phylogenetic data providing a relatively detailed view on the spread of at least two dengue viral lineages in the urban area of SJRP. These two groups suggested a pattern of dispersion that was consistent with the dispersal rate of *Aedes aegypti*, but in some instances, seemed to entail human accidental transport. It also showed that most of the cases until July traced directly to two viral sequences, sampled in January and February, respectively.

The connections among our samples averaged 3.75 kilometers, and the speed of propagation ranged from 0 to 428.8 meters per day. *Aedes aegypti* lays its eggs at many different sites (i.e., skip ovoposition) [39], maximizing spread potential and the chance of survival. Nevertheless, our data stressed the notion that both, mosquito and human circulation, do play an important role in the dispersal of the virus and were in good agreement with previous estimates of dispersion distances ranging from 15 to 800 meters [40],[41],[42]. The co-circulation of distinct dengue lineages in SJRP could be explained by independent introductions experiencing different dynamic outcomes or accretion of genetic diversity generated locally. Lineages can vanish from a given locality due to unfavourable conditions such as temperature, low mosquito population and changes in immunity status of the population during the outbreak. However, *Aedes aegypti* eggs are resistant to desiccation and this characteristic (*i.e.*, overwintering) may have important implications for the cryptic maintenance of viral strain. Therefore, viral lineages not detected in one season can re-emerge in the next rainy season due to transovarial transmission that is believed to be the most important factor for the maintenance of the virus in the nature [43]. Therefore it is not possible at this time to determine precisely the order of introduction events and the proper time of introduction of the distinct lineages that we have detected. The region of SJRP experiences an increase in rainfall starting in December reaching a peak in January/February. Concurrently, the number of dengue cases began to increase following the infestation by *Aedes aegypti*. In SJRP, the two main lineages were present at the zenith of dengue transmission, which coincided with the highest values for temperature and humidity. Another lineage (represented in green in Figures 4, 5, and 6) appeared later in May, when temperature, rainfall and humidity were decreasing. However, it is not known if this lineage became established (we could not identify putative links of this sample with other samples), given that the data collection finished in July 2006. Possibly viruses included in lineage 2 did not succeed in getting established in the city or faded away, given its appearance later in scene (June 6th) when the dryer and colder weather did not favour the development of the larvae and/or the survival and activity of the mosquito [44],[45]. Alternatively, its establishment may have been hampered by the decreasing availability of susceptible hosts in the later stages of the outbreak. The matrices of distances displayed in Figure 4 show how each sample is relatively close to all other samples, considering separately their date of collection, their place of collection and their genetics. As interesting they are in their own, each one cannot suggest a hypothetical scenario for the dynamics of the dispersion of dengue in São José do Rio Preto. Only when we combine all this information (time, space and genetics) based on parsimonious assumptions (each sample should be connected - by descended or siblinghood - to the closest genetic sample of the past) we can suggest a plausible dispersion scenario and, from this, infer other very useful information - like the speed or direction of the events.

DENV-3 was first isolated in São José do Rio Preto in January 2006. According to our results, both lineages split around one to three years before the collection of the last sample. In the four-year period at Figure 2, we noticed that during the three years after the introduction of DENV-2, in 1998, the incidence started to decrease every year, possibly due to the lack of susceptible hosts, but DENV-2 was still circulating in June 2005. The introduction of DENV-3 into the naive population to this serotype in SJRP may have occurred during 2005, because in September of this year, a neighborhood in the northern part of the city presented a significant increase in dengue incidences. The outbreak continued during October and November and culminated in April of 2006. The notion of a probable start of DENV-3 transmission at the Santa Clara neighborhood in September 2005 and its subsequent spread to the rest of the municipality (Figure 8) is in accordance with the results presented in Figures 2A and 3A. The outset of DENV-3 transmission at this underdeveloped urban area was to be expected, because *Aedes aegypti* larval infestation studies done in January 2005 [46] indicated that areas in SJRP with lower socioeconomic levels, with deficient sanitation infrastructure, presented higher infestation levels when compared to affluent neighborhoods inside the urban perimeter. The spread of dengue transmission through the entire municipality is in accordance with the high R_0_ values we found, possibly because the population was susceptible to serotype 3. An interesting result was that the BSL (Figure 2A), based on viral sequences recovered with great precision the dynamics of the epidemics obtained from case reports (Figure 2B). Nevertheless, the zenith determined by the BSL took place around February, two months before the maxima determined by case report in April (Figure 2). Interestingly, these results could be explained by the fact that up until March of 2006 clinical differential diagnostics was used in conjunction with serology, which increases the accuracy of the dengue diagnostics. On the other hand, after the number of cases exceeded 300 per 100,000 inhabitants in April 2006, only clinical criteria were used, which may have caused an increase in false positives, due to the lack of further serological confirmation. These results further validate the use of viral gene genealogies to infer epidemiological parameters of DENV in particular and, of fast-evolving viruses in general.

The use of home addresses for geo-positioning our patients for spatial analysis was justified because individuals spend considerable time at home, which constitutes a highly probable site of transmission. Nevertheless, transmission might also occur at other places and this fact certainly may have had some impact on our data, which would be hard to account for. Nevertheless, our exercise was valid, since it indicated coherent patterns of transmission, which may be relevant for implementing control measures. Our molecular data indicated that the viral spread was not dependent entirely on vector dispersal. The exponential growth phase associated with linked transmission events beyond the usual flight range of the mosquito may have been caused by under-sampling and movement of viremic humans, but is certainly indicative of fast transmission among susceptible individuals. Therefore, surveillance systems need to be capable of monitor proactively the occurrence of initial low levels of transmission, identify early cryptic circulation of new serotypes and, be able to map where infected patients are circulating, preferably at the lag phase of the outbreak.

Although we have not found clear relationships between dengue cases with molecular analysis and socioeconomic levels (Table 1 and Figure 9A), the first samples associated with the beginning of the outbreak, which were possible source nodes for many other samples, were found in regions with low socioeconomic level. Moreover, a higher transmission in poor areas of SJRP has been shown, especially in the north zones of the city [47]. Therefore, it is relevant to further evaluate if the occurrence of transmission in poor areas facilitates a higher dispersion of the virus to other areas of the city. However, the association between higher dengue transmission and low socioeconomic levels is controversial. Some studies have demonstrated the association of poverty and high incidences of dengue [48],[49], others have not [50],[51],[52] and others have indicated an inverse relation [53]. A study [48] demonstrated that dengue occurred at higher levels in poorer areas of SJRP in 1995 but, from 1998 to 2002, after the introduction of DENV-2, the variable that best explained dengue cases was the proportion of one-story homes. The socioeconomic features lost its explanatory power as the years passed and the spatial characteristic of the areas was more relevant [54]. A higher transmission of dengue in poor areas of the north zone was observed again in 2005–2006 [47].

Two hypotheses might explain the controversial pattern of dengue transmission. In both 1995 and 2006 epidemic season, dengue transmission started in poor northern zones of the city in the previous years (1994 and 2005) with a subsequent spread to other areas [12],[47],[48]. Therefore, the highest initial incidence of dengue in the north and the lowest in the other areas might be related to the usual delay in adopting of control measures at the beginning of the outbreak. Another hypothesis is that dengue transmission occurred initially in poor areas and spread to the rest of the city due to the reduction of susceptible individuals in the areas that were primarily affected and, as the years passed, the distribution of the disease became similar in the whole city. This pattern was confirmed previously [55], for the period of 1994 to 1998, when only DENV-1 was circulating in the city, but not for the period of 1998 to 2002, when DENV-1 and DENV-2 were circulating simultaneously.

The introduction of new DENV serotypes and genotypes constitutes a major risk factor for severe dengue manifestations [56]. But it is still controversial whether DENV strains that cause severe disease out-compete less virulent strains, which is a cause of major concern [22]. Therefore it is paramount to address in greater detail whether differences in viral dispersion patterns are associated with viral fitness, strain competition and, ultimately, whether it has any association with increase in disease severity. We have shown that spatial analysis using Geographic Information System could provide valuable information on dengue transmission and the spread of the disease in a defined but heterogeneous urban setting, typical of the developing world. We believe that the current study helped determining with greater precision areas where the infection took place, to understand particularities of an outbreak, clarifying the mechanisms of dengue transmission in SJRP. Ultimately, the association of molecular epidemiology with spatial analysis and the understanding of some biological and reproductive characteristics of *Aedes aegypti* mosquitoes may shed light on the dynamics and distribution of different dengue viral strains.
